# MRI performance to identify clinically significant prostate cancer following focal cryo‐ablation

**DOI:** 10.1002/bco2.70120

**Published:** 2025-12-10

**Authors:** Herbert Lepor, Samuel Parry, Majlinda Tafa, Eli Rapoport, James S. Wysock

**Affiliations:** ^1^ Department of Urology NYU Grossman School of Medicine New York NY USA

**Keywords:** ablation techniques, cryotherapy, intermediate risk prostate cancer, magnetic resonance imaging, prostate cancer

## Abstract

**Objectives:**

To determine the performance of MRI for detecting clinically significant prostate cancer (csPCa) recurrence following focal cryo‐ablation over five years of oncological surveillance.

**Subjects and Methods:**

A total of 305 men with intermediate‐risk prostate cancer undergoing focal cryo‐ablation (FCA) at our referral centre between 03/2017 and 03/2024 were prospectively enrolled in an outcomes registry. Selection criteria, treatment planning and oncological surveillance were standardized. The initial surveillance protocol included MRI at six months, two, 3.5, five years, and surveillance prostate biopsy at six months, two and five years. The surveillance protocol biopsy at six months and two years was abandoned for a negative MRI in May 2019 and November 2020. Performance statistics of MRI for detecting csPCa recurrence were assessed among mandatory MRI and biopsy dyads at six months and two years. Clinically significant prostate cancer detection rates for suspicious (positive) and negative MRI included all subjects.

**Results:**

Sensitivity, specificity, positive predictive value and negative predictive value of MRI to predict csPCa is 25%, 93%, 29% and 91%. The csPCa detection rate for positive and negative MRI was 40% and 7.0%, respectively. The area under the receiver operating characteristic curve for MRI as a predictor of csPCa was 0.60, suggesting limitations of MRI for predicting csPCa. A limitation is that a validated instrument for positive MRI was not available during the study.

**Conclusions:**

Six‐month MRI is not recommended owing to its very low csPCa detection rate. The increasing csPCa detection rates at two and five years suggest these are reasonable time points to perform MRI. Prostate biopsy should be performed on all cases with positive MRI, and can safely be deferred in most cases with negative MRI.

## 1 INTRODUCTION

The American Urological Association and European Urological Association guidelines recommend counselling men with intermediate‐risk prostate cancer that focal therapy (FT) is experimental, and men pursuing FT should be followed as part of a clinical trial.^1^, ^2^

MRI imaging coupled with targeted and systematic biopsy has enabled the selection of candidates for FT.^3^ FT in selected cases of csPCa achieves comparable intermediate‐term oncological control with fewer treatment‐related complications and superior functional outcomes compared to radical prostatectomy.^4^, ^5^

All men undergoing treatment for localized prostate cancer must undergo surveillance for disease recurrence. Since whole‐gland treatment removes or eradicates the entire gland, disease recurrence is initially monitored with PSA testing alone. Additional testing is recommended when the PSA reaches a level indicative of biochemical recurrence defined as a post‐prostatectomy PSA > 0.2 ng/ml or PSA nadir + 2 ng/ml following radiation therapy.^6^, ^7^

Assessing disease recurrence following FT is challenging since a significant component of the prostate is intentionally preserved in order to optimize functional outcomes. There is no consensus regarding the timing of MRI or indications for prostate biopsy beyond one year of treatment, and what constitutes a suspicious MRI that justifies prostate biopsy.^8^, ^9^, ^10^

Since March 2017, men undergoing FCA provided informed consent to participate in an IRB‐approved prospective longitudinal outcomes registry. The study has been performed according to the Declaration of Helsinki. MRI was performed at specific time points, and indications for prostate biopsy and the biopsy protocol were prospectively defined and standardized. The objective of the present study is to determine the performance of MRI for detecting csPCa recurrence following FCA. Our study provides evidence‐based recommendations for the timing of MRI and its performance for predicting csPCa recurrence following FCA.

## 2 SUBJECTS AND METHODS

### 2.1 Subjects

Our prospective IRB approved outcomes registry following FCA was initiated in March 2017 (IRB #17–00354). Patient selection has been previously reported.^11^, ^12^ The present analysis included only subjects with intermediate‐risk disease with an MRI PI‐RADS 2–5 lesion concordant with unilateral intermediate‐risk disease (Gleason Group [GG] 2 or 3 disease), no gross extra‐prostatic extension on MRI, no GG ≥ 2 contralateral to the target and no very distal apical disease on MRI.

### 2.2 Treatment

All FCA were performed under general anaesthesia in the dorsal lithotomy position using the Cryocare CS® system as previously described.^11^, ^12^

### 2.3 Surveillance Protocol

The initial surveillance protocol included PSA testing at 3 and 6 months following FCA and every 6 months thereafter, an MRI at 6 months, 2 years, 3.5 years and 5 years, and a surveillance prostate biopsy at 6 months, 2 years and 5 years. All MRI studies included T2 weighted imaging (T2W), dynamic contrast enhancement (DCE) and diffusion‐weighted imaging (DWI) sequences. The surveillance protocol biopsy independent of MRI findings at 6 months and 2 years was abandoned in May 2019 and November 2020, respectively, following interim analysis demonstrating very low csPCa detection rates for negative MRI.^11^, ^12^ In addition to this routine surveillance, we currently advise MRI and biopsy at any time point if DRE, PSA or MRI is suspicious for csPCa. We consider the MRI suspicious for out of field csPCa recurrence (positive MRI) if a new out‐of‐field PI‐RADS >2 lesion is observed, or a previously observed MRI lesion increases in PI‐RADS score or maximal linear diameter. A positive in‐field MRI was based on early in‐field contrast enhancement or a persistent or new diffusion abnormality. Based on the post‐treatment MRI, the radiologist commented whether the study was suspicious for in‐field recurrence. Additionally, given that prior analysis demonstrated contralateral GG = 1 at baseline is predictive of csPCa at 3‐year follow‐up,^12^ these men were advised to undergo biopsy at 2 and 5 years regardless of DRE, PSA or MRI.

The post FCA biopsy protocol included 4 cores directed into the ablation zone, 4 cores directed into any suspicious in‐ or out‐of‐field MRI targets and a 12‐core computer‐generated systematic biopsy (SB).

### 2.4 Outcomes

Between March 2017 and May 2019, 53 men were advised to undergo protocol MRI and prostate biopsy at 6 months independent of PSA, DRE or MRI; 79% of the subjects were compliant with the surveillance protocol (i.e., underwent both MRI and prostate biopsy prior to 1 year). Since November 2018, 238 additional subjects were eligible for 6‐month oncological surveillance and biopsies were recommended for positive MRI or at the discretion of the surgeon.

Between September 2018 and November 2020, 53 additional subjects were advised to undergo protocol MRI and prostate biopsy at 2 years independent of PSA, DRE or MRI; 66% of the subjects were compliant with the surveillance protocol (i.e., underwent both MRI and prostate biopsy 1–2.5 years following treatment). Since November 2020, 144 subjects were eligible for 2‐year oncological surveillance. Biopsies were recommended if there was a positive MRI, identification of contralateral low‐risk disease on prior biopsy, or at the discretion of the surgeon.

Between September 2021 and June 2024, 78 men were advised to undergo protocol MRI at 5 years. Eighty‐eight percent of the subjects were compliant with the surveillance protocol (i.e., underwent MRI 4–6 years following treatment). Biopsies were recommended for positive MRI, identification of contralateral low‐risk disease on prior biopsy, or at the discretion of the surgeon.

### 2.5 Statistical Analysis

The performance statistics of MRI for detecting post‐treatment csPCa were ascertained only for those subjects who were compliant with the 6‐month and 2‐year surveillance protocol, which at the time included MRI and prostate biopsy in all subjects. Sensitivity, specificity, PPV and NPV of MRI were evaluated in a pooled sample of all 6‐month and 2‐year MRI and biopsy dyads. The area under the receiver operating characteristic curve (ROC AUC) was calculated for MRI as a predictor of csPCa at PI‐RADS scores 1–5 as the threshold for positive MRI. Analyses were performed using R, version 4.3.2, between June 2024 and September 2024.

## 3 RESULTS

A total of 305 subjects with intermediate‐risk disease meeting the study criteria underwent FCA between March 2017 and March 2024. The baseline demographics, radiological and pathological characteristics are summarized in Table 1.

**TABLE 1:** Baseline characteristics of study cohort (n = 305).

Demographics and Baseline Characteristics	No. (%)
Race	
Black	36 (11.8%)
White	235 (77.0%)
Other/Multiracial	34 (11.2%)
Ethnicity	
Hispanic	26 (8.5%)
Non‐Hispanic	279 (91.5%)
PI‐RADS^a^	
2	24 (7.9%)
3	90 (29.5%)
4	149 (48.9%)
5	40 (13.1%)
Gleason Grade Group (GGG)	
2	220 (72.1%)
3	85 (27.9%)
Out‐of‐field GGG 1 lesion	87 (28.5%)
	Median (IQR)
Age, y	65.0 (61,70)
Prostate volume, mL	43 (30.9, 59.1)
PSA, ng/mL	7.39 (4.51, 8.25)
PSA density, ng/mL^2^	0.14 (0.09, 0.20)
Maximum dimension of index lesion, mm	12 (8,15)

^a^ Two PI‐RADS scores were not assigned due to artefact from a metallic object in the pelvis.

### 3.1 Six Month Surveillance

**FIGURE 1:**
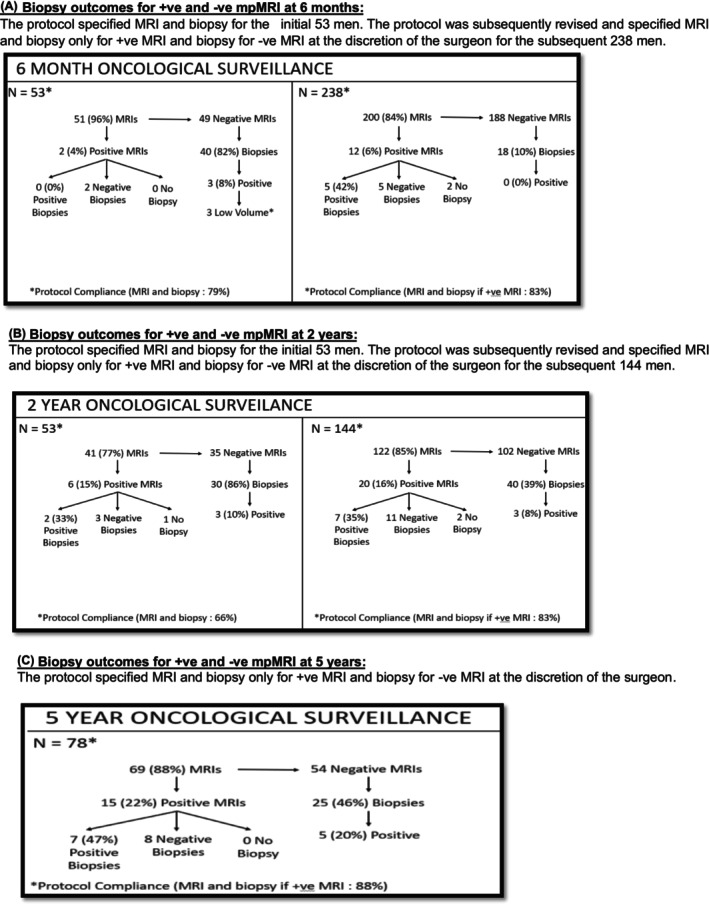
(A) Biopsy outcomes for +ve and ‐ve mpMRI at 6 months: The protocol specified MRI and biopsy for the initial 53 men. The protocol was subsequently revised and specified MRI and biopsy only for +ve MRI and biopsy for ‐ve MRI at the discretion of the surgeon for the subsequent 238 men. (B) Biopsy outcomes for +ve and ‐ve mpMRI at 2 years: The protocol specified MRI and biopsy for the initial 53 men. The protocol was subsequently revised and specified MRI and biopsy only for +ve MRI and biopsy for ‐ve MRI at the discretion of the surgeon for the subsequent 144 men. (C) Biopsy outcomes for +ve and ‐ve mpMRI at 5 years: The protocol specified MRI and biopsy only for +ve MRI and biopsy for ‐ve MRI at the discretion of the surgeon.

A total of 291 subjects were eligible for 6‐month csPCa surveillance (Figure 1A). Fifty‐three of these subjects were enrolled when our surveillance protocol stipulated an MRI and prostate biopsy on all subjects at 6 months. Of the 51 (96%) subjects undergoing a protocol MRI, a positive MRI was observed in 2 (4%) men, both of whom had negative biopsies. Of the 49 negative MRIs, 40 (82%) underwent a prostate biopsy and 3 (8%) exhibited csPCa. Based on both the low rates of abnormal MRIs and csPCa detection rates associated with negative MRIs, we abandoned protocol surveillance biopsy at 6 months for all subjects.^11^ Since then, 200 (84%) of 238 eligible subjects underwent a protocol 6‐month MRI. Of these 200 MRIs, 12 (6%) were positive, of whom 10 underwent recommended biopsy; 5 (50%) exhibited csPCa on biopsy. Of the 188 negative MRIs, 18 underwent prostate biopsy and none exhibited csPCa. The five (1.7%) men exhibiting csPCa on the 6‐month biopsy were censored at this time point.

### 3.2 Two Year Surveillance

A total of 197 subjects were evaluable for 2‐year csPCa surveillance (Figure 1B). Fifty‐three of these subjects were enrolled when the surveillance protocol stipulated a 2‐year MRI and prostate biopsy for all subjects. 41 (77%) of these men underwent a protocol MRI. MRI was positive in 6 (15%) cases, of whom one refused prostate biopsy and 2/5 (40%) had biopsies exhibiting csPCa. Of the 35 negative MRIs, 30 (86%) underwent a prostate biopsy and 3 (10%) exhibited csPCa. Based on the low csPCa detection rate in men with negative MRIs, we abandoned protocol surveillance biopsy at 2 years.^12^ Since then, 122 (85%) of 144 eligible subjects underwent a protocol 2‐year MRI. Of these 122 MRIs, 20 (16%) were positive of whom two refused prostate biopsy and 7 of 18 (39%) exhibited csPCa. Of the 102 negative MRIs, 40 underwent prostate biopsy and csPCa was detected in 3 (8%). The 9 (4.6%) men exhibiting csPCa on the 2‐year biopsy were censored at this time point.

### 3.3 Five Year Surveillance

A total of 78 subjects were eligible for 5‐year oncological surveillance (Figure 1C). Based on prior experiences, our surveillance protocol stipulated an MRI and subsequent biopsy only for positive MRI or at the discretion of the surgeon. Of the 78 eligible subjects, 69 (88%) underwent a protocol MRI. Of these 69 5‐year MRIs, 15 (22%) were positive, all of whom underwent biopsy. Seven of 15 (47%) exhibited csPCa. Of the 25 (46.3%) of 54 subjects with negative MRI, who underwent prostate biopsy, csPCa was detected in 5 (20%).

### 3.4 mpMRI Performance

**FIGURE 2:**
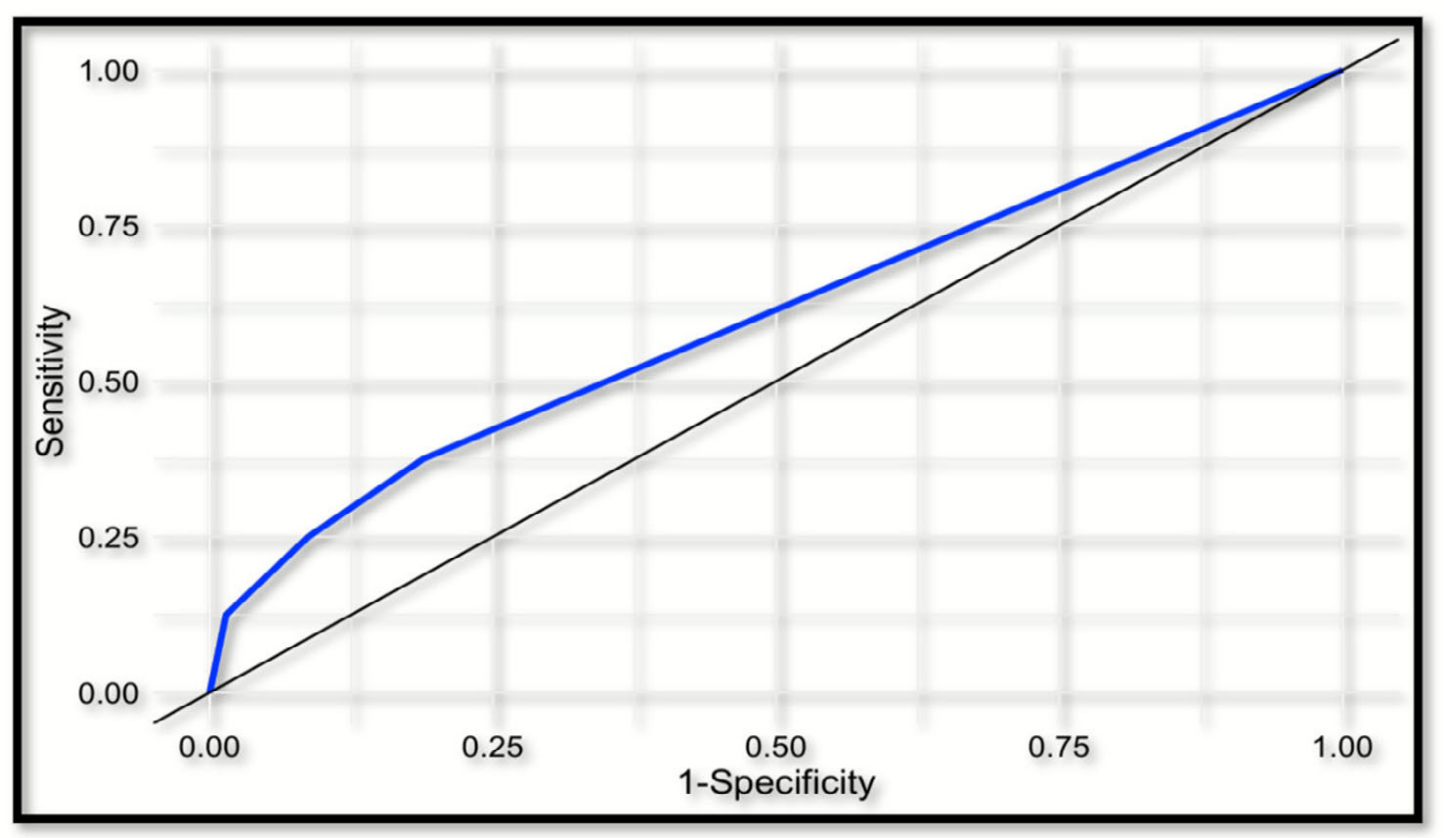
Receiver operator characteristics (ROC) curve of mpMRI for predicting csPCa recurrence following PPGCA.

The sensitivity, specificity, positive predictive value and negative predictive value for csPCa for the pooled 6‐month and 2‐year outcomes when biopsy was recommended in all men undergoing MRI is 25%, 93%, 29% and 91% (Table 2). The ROC AUC (Figure 2) for MRI as a predictor of csPCa at PI‐RADS scores of 1–5 as a threshold for a positive MRI was 0.60.

**TABLE 2:** Performance of mpMRI for detecting csPCa.

	Positive Biopsy	Negative Biopsy	
Positive MRI	2	5	PPV: 29%
Negative MRI	6	64	NPV: 91%
	Sensitivity: 25%	Specificity: 93%	

When protocol biopsy was performed at 6 months and 2 years independent of MRI findings, the csPCa detection rate in 70 subjects with a negative MRI was 8.6%. Once protocol biopsy was performed for positive MRI or at the discretion of the surgeon, the csPCa detection rate in 57 subjects with negative MRI was only 4.7%. The csPCa detection rate in men with positive MRI and negative MRI was 40% and 7%, respectively.

## 4 DISCUSSION

There is increasing evidence that FT is a reasonable treatment option for men with focal intermediate‐risk prostate cancer based on oncological and functional outcomes.^13^ The primary limitation of FT is the potential consequences of local disease recurrences progressing to metastasis and subsequent prostate cancer mortality. It is therefore imperative to define an evidence‐based surveillance protocol for detecting csPCa recurrences following FT. While there is consensus that monitoring following FT should include serum PSA and MRI, the timing of testing and indications for prostate biopsy have not been defined.^8^, ^9^, ^10^

Since March 2027, we have enrolled over 500 subjects undergoing FCA by two urologic oncological surgeons (HL and JW) into an IRB‐approved outcomes registry. A unique strength of our outcomes registry is that a rigorous oncological surveillance protocol was prospectively defined and followed with excellent compliance. While focal cryo‐ablation was performed in select cases of low‐ and high‐risk disease, we report on only the 305 men with intermediate‐risk disease.

The primary goal of the present study was to examine the role of MRI for detecting csPCa recurrence following FCA. We defined csPCa as any Gleason pattern 4 on prostate biopsy.^14^ We employed a rigorous biopsy protocol, which included 4 tissue cores into the ablation zone, 4 tissue cores into any suspicious mpMI target and 12 computer‐generated SB. We considered a positive out‐of‐field MRI as any interval growth in existing PI‐RADS lesions, increased PI‐RADS scoring or new PI‐RADS >2 target. A positive in‐field MRI was based on early in‐field contrast enhancement or a persistent or new diffusion abnormality. Based on the post‐treatment MRI, the radiologist commented whether the study was suspicious for in‐field recurrence. The performance of MRI for detecting csPCa recurrence can only be defined if all subjects undergo a prostate biopsy independent of MRI findings. A total of 106 subjects in our cohort were advised to undergo protocol MRI and prostate biopsy at 6 months and 24 months, respectively. Our compliance for undergoing 6‐month and 2‐year protocol MRI was 96% and 77%; compliance for protocol MRI and biopsy was 79% and 66%, respectively. Combining these two points, the sensitivity, specificity, PPV and NPV of MRI for detecting csPCa were 25%, 93%, 29% and 91%. Based on the consistently high NPV of MRI, we abandoned performing protocol biopsy on all subjects with negative MRI at all‐time points. The AUC of 0.60 highlights limitations of MRI to detect csPCa recurrence following focal cryo‐ablation, primarily due to poor sensitivity.

Our compliance with a 5‐year MRI is 88% and the rate of positive MRI was 20%, which is higher than 2 years. Our overall csPCa detection rate in the subset with negative MRI at 5 years was 13%. Of the 46.3% of negative MRIs undergoing prostate biopsy, csPCa was detected in 20%. Presumably, the subset of men undergoing biopsy represented selection bias for those at risk for csPCa recurrence according to the discretion of the surgeon.

Since adopting our revised oncological surveillance protocol, 238 and 144 additional men were eligible for 6‐month and 24‐month follow‐up. Our compliance rate was 83% with MRI at 6 and 24 months.

The decision to perform a prostate biopsy following FT should take into consideration the morbidity of biopsy and detection rates of csPCa. In our composite outcomes registry, the rates of positive MRI were 6%, 16% and 22% at 6 months, 2 years and 5 years, respectively. The rates of csPCa for positive MRI at 6 months, 2 years and 5 years were 42%, 39% and 47%. The overall 42% csPCa detection rate in positive MRI justifies performing biopsy in these men. The rates of negative MRI in our composite cohort at 6 months, 2 years and 5 years were 94%, 84% and 78%, respectively. The csPCa detection rate in subjects with a negative MRI at 6 months, 2 years and 5 years was 5%, 9% and 20%, respectively. The overall low csPCa detection rate in negative MRI justifies our decision not to biopsy these men at any time point.

It is imperative to balance oncological and functional outcomes. We have achieved these encouraging oncological outcomes while also maintaining excellent functional outcomes.^15^, ^16^

A systematic review and consensus statement were conducted by 23 panellists representing thought leaders from Europe and North America in urology, radiology and pathology. The reported transatlantic recommendations for prostate gland evaluation with magnetic resonance imaging after focal therapy (TARGET) were based on the systematic review and consensus study.^10^ Because of the absence of high‐quality studies, recommendations were based on consensus. The recommendation for the timing of the first MRI was 1 year since in‐field DCE prior to 1 year might represent granulation tissue or disease recurrence. The literature review did not provide evidence supporting the required MRI sequences and criteria constituting a suspicious MRI requiring post‐treatment biopsy. The consensus was to perform MRI with T2w, DCE and DWI sequences and define the risk of in‐field recurrence using the TARGET scoring system, which emphasizes DCE but also considers T2W and DWI findings. The group emphasized that the TARGET score has not been validated based on a large cohort of biopsies performed independent of the level of suspicion for in ‐field disease recurrence.

The expert group made several suggestions for future investigation. They emphasized the need to report studies from institutions with surgeons and radiologists experienced in performing FT and interpreting MRI following FT. Since 2012, surgeons at our institution have performed over 1000 FTs and our radiologists are thought leaders in the field of prostate MRI. A reported limitation of the literature was the lack of any study performing MRI biopsy independent of MRI findings on a consecutive cohort of men undergoing FT. In the present study, 106 men underwent prostate biopsy independent of MRI findings, which informed our decision to abandon routine prostate biopsy for negative MRI. Another reported limitation of the literature was the lack of MRI and biopsy outcomes beyond 1 year of follow‐up. In the present study, we report a large cohort of men at 2 and 5 years.

There are many other strengths of the present study. Only men with intermediate‐risk cancer were investigated since this defines the most appropriate candidates for FT.^8^, ^9^ Patient selection, treatment planning and surveillance protocol were defined prior to enrolment in the study and only revised based on our outcomes. Definitions of csPCa and positive MRI were consistent throughout follow‐up. The biopsy protocol involved rigorous tissue sampling even in those men with negative MRI. A large cohort of men at 6 and 24 months underwent protocol MRI and prostate biopsy, which enabled investigating the performance of MRI to detect csPCa following FCA. Our study also includes a large validation cohort that confirmed a low csPCa detection rate in subjects with negative MRI who were selected for biopsy and reinforced our decision to abandon routine biopsy of negative MRIs.

There are notable limitations to the present study. The performance of MRI for detection of csPCa is dependent on csPCa detection rates, which in our study were quite low. MRI may have different performance characteristics in cohorts with higher csPCa detection rates. Notably, our results cannot be generalized to other ablative energy sources since the confluence of energy delivery may vary according to energy sources.

Another limitation of our study is that we did not use a quantitative scoring system to assess the risk of in‐field recurrence. In 2017 when we initiated our study, there was no consensus regarding an instrument to assess in‐field disease recurrence. Giganti et.al^17^ has recently proposed a three‐point MRI scoring system, PI‐FAB for assessing disease recurrence following FT based on dynamic contrast enhancement and b‐value‐diffusion weighted sequences. This scoring system was not validated by performing biopsy on all cases with PI‐FAB scores. The TARGET score also has not been validated but by consensus it is a recommended instrument for assessing the risk of in‐field disease recurrence following FT. In the present study, our radiologists relied on all sequences to report qualitatively whether the MRI was suspicious for disease recurrence without a numerical rating. While we plan to adopt the TARGET score as we further explore timing and indications for MRI and prostate biopsy following FT, our study represents the most rigorous assessment of MRI and the risk of disease recurrence following FT reported in the literature.

Our study supports utilizing MRI to inform decisions to perform prostate biopsy following FT. Our study suggests that the 6‐month MRI may be abandoned due to very low rates of positive MRIs and overall csPCa detection rates. We recommend surgeons first confirm low rates of positive MRI at 6 months prior to abandoning testing at this time point. The increasing rates of positive MRI and csPCa detection rates at 2 and 5 years suggest these are reasonable time points to perform MRI. The low csPCa detection rate in men with negative MRI suggests biopsy in these men can be safely deferred.

## AUTHOR CONTRIBUTIONS

**Herbert Lepor:** Conception and design, analysis and data interpretation, draft preparation, critical revision of the manuscript, supervision. **Samuel Parry:** Acquisition of data, analysis and data interpretation, critical revision of the manuscript, statistical analysis. **Majlinda Tafa:** Acquisition of data, analysis and data interpretation, critical revision of the manuscript. **Eli Rapoport:** Analysis and data interpretation, critical revision of the manuscript, statistical analysis. **James S. Wysock:** Analysis and data interpretation, critical revision of the manuscript.

## CONFLICT OF INTEREST STATEMENT

The authors declare that they have no known competing financial interests or personal relationships that could have appeared to influence the work reported in this paper.

## Data Availability

The datasets generated during and/or analysed during the current study are not publicly available to maintain participant privacy, but are available in deidentified form from the corresponding author on reasonable request.
